# Neuroprotective Role of the PI3 Kinase/Akt Signaling Pathway in Zebrafish

**DOI:** 10.3389/fendo.2017.00021

**Published:** 2017-02-08

**Authors:** Shuang Chen, Yunzhang Liu, Xiaozhi Rong, Yun Li, Jianfeng Zhou, Ling Lu

**Affiliations:** ^1^Key Laboratory of Marine Drugs (Ocean University of China), Chinese Ministry of Education, School of Medicine and Pharmacy, Ocean University of China, Qingdao, Shandong, China

**Keywords:** PI3 kinase, Akt, IGF, zebrafish, brain

## Abstract

Neuronal survival and growth in the embryo is controlled partly by trophic factors. For most trophic factors (such as Insulin-like growth factor-1), the ability to regulate cell survival has been attributed to the phosphoinositide 3-kinase (PI3K)/Akt kinase cascade. This study presents data illustrating the role of PI3K/Akt in attainment of normal brain size during zebrafish embryogenesis. Blocking PI3K with inhibitor LY294002 caused a significant reduction in brain size (in addition to global growth retardation) during zebrafish embryogenesis. This PI3 Kinase inhibition-induced brain size decrease was recovered by the overexpression of myristoylated Akt (myr-Akt), a constitutive form of Akt. Further analysis reveals that expressing exogenous myr-Akt significantly augmented brain size. Whole mount *in situ* hybridization analysis of several marker genes showed that myr-Akt overexpression did not alter brain patterning. Furthermore, the expression of myr-Akt was found to protect neuronal cells from apoptosis induced by heat shock and UV light, suggesting that inhibition of neuronal cell death may be part of the underlying cause of the increased brain size. These data provide a foundation for addressing the role of PI3K/Akt in brain growth during zebrafish embryogenesis.

## Introduction

Neural cell survival and death are precisely controlled in animal early developmental stage (1). During nervous system development, approximately half of all neurons are eliminated by apoptosis. Besides sculpting the developing brain, neuronal apoptosis has a critical role in many neurological diseases, such as Alzheimer’s, Parkinson’s, and Huntington’s disease (2). Neuronal death can be regulated and suppressed by a variety of growth factors. For example, normal brain growth requires insulin-like growth factor 1 (IGF1), and IGF1 deficiency decreases brain size by reducing both cell number and cell size (3–5). Additionally, targeted deletion of IRS2, the major downstream IGF1 receptor, also produced a pronounced brain growth deficiency in mice, in which the reduction became apparent during embryonic (E15.5) development (6). Furthermore, overexpressing IGF1 led to an increase in mice brain mass (7).

In the past decade, the ability of growth factors to promote neuronal survival has been attributed, at least in part, to the phosphoinositide 3-kinase (PI3K)/Akt pathway (8, 9). PI3K stimulates the activation of Akt/protein kinase B (Akt/PKB), and this activation appears to be one of the most important steps for regulating cell survival, cell size, and proliferation (10, 11). In mammals, there are three Akt isoforms: Akt1 (*PKB*α), Akt2 (*PKB*β), and Akt3 (*PKB*γ), which are highly conserved among species. Akt1 deficiency resulted in significant neonatal mortality and growth retardation due to a defect in placental development (12–14). Akt2^−/−^ mice displayed insulin resistance and a type-II diabetes-like syndrome, accompanied with mild growth retardation and age-dependent loss of adipose tissue (15). Akt3^−/−^ exhibited an approximate 20% reduction in brain size and weight in adult mice due to decreases in cell size and number, which indicates the importance of Akt3 in brain development (16). Furthermore, Akt1/2 and Akt2/3 double knockout mice both exhibited more severe defects in development and survival than single knockout mice, reinforcing the compensatory actions and functional essentiality of Akt proteins during development (17, 18).

On the contrary, overexpression studies revealed that Akt1 provides neuroprotection to human neuronal cells (hNSCs) under the conditions of oxidative stress *in vitro* and improves hNSCs survival and brain function recovery of mouse intracerebral hemorrhage stroke model (19). Research also showed that Akt1 function enhanced neuronal cell survival *in vitro* and *in vivo* (20, 21), whereas Akt2 was found to be able to protect the retina from light-stress (22). Together, these studies illustrate an important requirement of Akt in the promotion of neuron growth and survival, which are also essential for brain growth.

Despite current progresses, the precise functions of Akt in early brain embryogenesis are incompletely understood due to *in utero* development of mouse. In contrast, the zebrafish embryo is ectogenesis and transparent at early developmental stages, which is an ideal alternative model system for studying the embryonic function of genes and proteins. Several distinct zebrafish Akt proteins have been identified: zebrafish Akt1, Akt2, Akt2-like, Akt3a, and Akt3b (ZFIN: The Zebrafish Model Organism Database). Their structures are highly conserved with Akt proteins in other vertebrate species, suggesting the similar functional roles they might play in zebrafish.

In this study, we set out to analyze functions of PI3K/Akt during zebrafish embryogenesis. We observed that inhibiting PI3K in zebrafish embryos with LY294002 resulted in decrease of brain size significantly. Those phenotypes could be recovered by overexpressing mouse myristoylated Akt (myr-Akt) *via* mRNA injection at early stage of zebrafish embryogenesis. Overexpression of myr-Akt alone increased brain size significantly at 24 h post fertilization (hpf) while having no effects on brain patterning. Last, we showed that constitutive activation of Akt protected developing zebrafish neuronal cells from apoptosis induced by heatshock and UV exposure. These results provide a foundation for future work addressing the embryonic functions of Akt in the zebrafish.

## Materials and Methods

### Experimental Animals

Adult wild-type zebrafish (*Danio rerio*) were maintained at 28°C on a 14 h:10 h (light:dark) cycle, and fed twice daily. Embryos were generated from natural crosses. Fertilized eggs were raised in embryo medium at 28.5°C and staged according to Kimmel et al. (23). This study was carried out in accordance with the recommendations of the Animal Research and Ethics Committee of Ocean University of China. The protocol was approved by the Animal Research and Ethics Committee of Ocean University of China.

All chemicals and reagents were purchased from Fisher Scientific (Pittsburgh, PA, USA), unless otherwise noted. Restriction endonucleases were purchased from New England BioLabs (Beverly, MA, USA). The anti-Akt and anti-Phospho-Akt antibodies (Ser473) and the anti-p70s6 kinase and anti-Phospho-p70s6 kinase (Ser411) antibodies were purchased from Cell Signaling (Danvers, MA, USA).

### Synthesis and Microinjection of mRNA

Capped mRNA synthesis was carried out using a commercial kit using mouse myr-Akt1 and GFP-linearized plasmid DNA (linearized with *Not*I digests) as template (Megascript kit; Ambion Inc., Austin, TX, USA). mRNA were injected into 1–2 cell embryos as previously reported (24). As a control, we injected mRNA encoding GFP at a concentration of 200 pg per embryo. After injection, embryos were placed in embryo rearing medium (5 mM NaCl, 0.17 mM KCl, 0.33 mM CaCl_2_, and 0.33 mM MgSO_4_) and kept at 28.5°C.

### *In Situ* Hybridization Analysis

Whole mount *in situ* hybridization using digoxigenin-labeled RNA riboprobes was carried out as reported previously (25).

### TUNEL Assays

Embryos were injected with 200 pg of either GFP mRNA (control) or mouse myr-Akt1 mRNA. At 20 hpf, 50 embryos in each treatment group were subjected to either heatshock (10 min incubation in a dry-air 37°C incubator in 50 ml of embryo rearing solution) or UV light treatment (60 s in a UV cross linker set at 100 mJ/cm^2^ in 5 ml of embryo rearing solution). Embryos were then transferred to non-treated embryo rearing solution and allowed to incubate for 8 h, at which they were fixed in 4% paraformaldehyde for later analysis.

Embryo embedding, freezing, and sectioning protocols were performed according to Brunet et al. (26). Sections (10 µm) were collected and air dried at room temperature for 2 h before staining or storage at −20°C. For TUNEL assays, sections were stained using the *In Situ* Cell Death Detection Kit, TMR Red, according to the manufacturer’s instructions (Roche, Nutlet, NJ, USA). Nuclei were counterstained with 50 nM Sytox (Molecular Probes, Carlsbad, CA, USA). Images of TUNEL and Sytox staining were captured separately and then merged using Adobe Photoshop. Quantification of TUNEL-positive cells and total cell number (Sytox) was done using the particle analysis function of ImageJ. All thresholds were set and used for the analysis of every image. A total of six images for TUNEL and Sytox staining were quantified per treatment group.

### Drug Treatment

The PI3K inhibitor, LY294002, was dissolved in dimethyl sulfoxide (Amresco, Solon, OH, USA) as a 50 mM stock solution and then added to embryo rearing medium; the final concentration of LY294002 was 8, 16, or 32 µM according to different experiments. For treatment of PI3K inhibitor, embryos at 10 hpf were transferred into embryo rearing medium containing different concentrations of LY294002, and then washed out and dechorionated at 24 hpf for inspection.

### Acridine Orange Staining

Dechorionate embryos and place in 5 µg/ml acridine orange (Sigma, USA) in embryo rearing medium. After 30 min of staining, wash the embryos twice with embryo rearing medium and view using fluorescence microscope with green filter. Quantification of apoptosis cells was done using the particle analysis function of ImageJ 1.41 (NIH, USA). All thresholds were set and used for the analysis of every image.

### Western Immunoblots

Twenty-five embryos from each treatment group were dechorionated, deyolked, and homogenized in 25 µl of RIPA buffer (50 mM Tris–HCl, 150 mM NaCl, 2 mM EGTA, 0.1% Triton X-100, pH 7.5) containing 10 µg/ml aprotinin, 10 μg/ml leupeptin, 10 µg/ml pepstatin, 100 mM PMSF, and 0.1 M sodium orthovanadate. The homogenates were briefly centrifuged to pellet cellular debris and the supernatant was retained. Each sample was subjected to SDS-PAGE (12.0%) and transferred to Immoblin-P membrane (Millipore Corp., Billerica, MA, USA). The total Akt antibody and the phospho-Akt antibody (Ser473) were used at a 1:1,000 and 1:2,000 dilutions, respectively.

### Brain Measurements

Brain sizes were determined at 24 hpf by measuring the linear distance from the forebrain to the mid–hindbrain boundary, bisecting the lens (called the horizontal brain length, H), and by measuring the linear distance from the yolk sac to the top of the brain, bisecting the lens (called the vertical brain height, V). Each value was then placed over embryo length, which is the curvilinear distance from the forebrain and midbrain boundary to the tail (L) unless otherwise noted. This calculation is called relative brain height (H/L) and relative brain length (V/L) and was determined with the ImageJ 1.41 (NIH, USA) for each embryo from pictures. Each line was then measured using a scale taken at the same magnification.

### Statistics

Data are presented as means ± SE. Differences among groups were analyzed by One-Way ANOVA followed by Fisher *Post hoc* Test or *t*-test (SPSS Inc., Chicago, IL, USA). Significance was accepted at *P* < 0.05.

## Results

### Inhibition of PI3 Kinase Leads to Decrease of Brain Size

**Figure 1:**
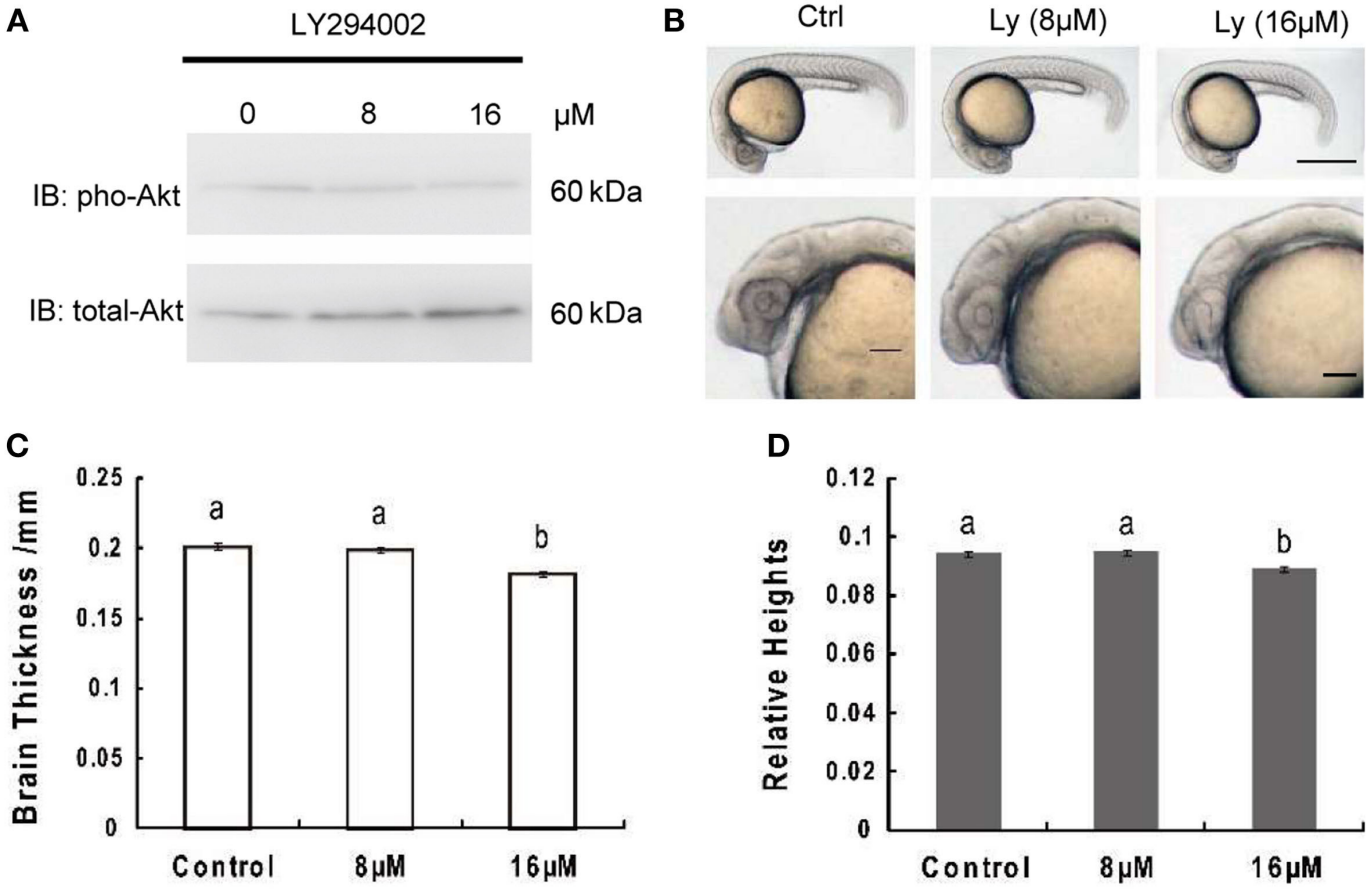
**Inhibition of phosphoinositide 3-kinase leads to decrease of brain size significantly over growth retardation**. **(A)** Representative Western immunoblots using antibodies for phosphorylated and total Akt on embryos treated with 8 μM, 16 μM, or without LY294002. Note the increase in total levels of Akt and gradually decreased phosphorylated Akt in LY294002 treated embryos compared to controls. Similar results were obtained in three other treatment experiments. **(B)** Morphology of 24 hpf embryos of wild type and treated with 8 µM, 16 µM LY294002. The top panel, scale bar = 500 μm; the bottom panel shows a close-up of the head region, scale bar = 200 μm. **(C)** Brain thickness and **(D)** relative heights of embryos from control group and embryos treated with 8 µM, 16 µM LY294002. Results are from three independent microinjection experiments, each with 30 embryos per group. *P* < 0.05 compared with the control group.

Akt phosphorylation levels at 24 hpf were obviously attenuated in zebrafish embryos treated with LY294002, while total Akt levels remained unchanged (Figure 1A). Meanwhile, embryos with 8 or 16 µM LY294002 treatment at 24 hpf displayed an overall reduction of body length and an uncharacteristic cloudiness or opacity in the head region (Figure 1B). However, the trunk region appeared to be relatively normal. We also observed other phenotypes commonly associated with PI3K-treated embryos, such as hypoplasia of the yolk sac extension, and delayed pigmentation. On close-up examination of those embryos, we determined significant reduction of brain thickness (linear distance form yolk sac to top of head, bisecting the lens) as that found in IGF1R knockdown research (Figure 1C); and the relative brain thickness (expressing brain thickness over whole embryo length, which is the linear distance from inner forehead to tail) also displayed an obvious trend of decrease (Figure 1D). It elucidated that the brain exhibited more severe size reduction over the whole retardation of growth.

### Overexpression of myr-Akt Increase the Levels of Phosphorylated Akt and p70S6 Kinase in Zebrafish Embryos

**Figure 2:**
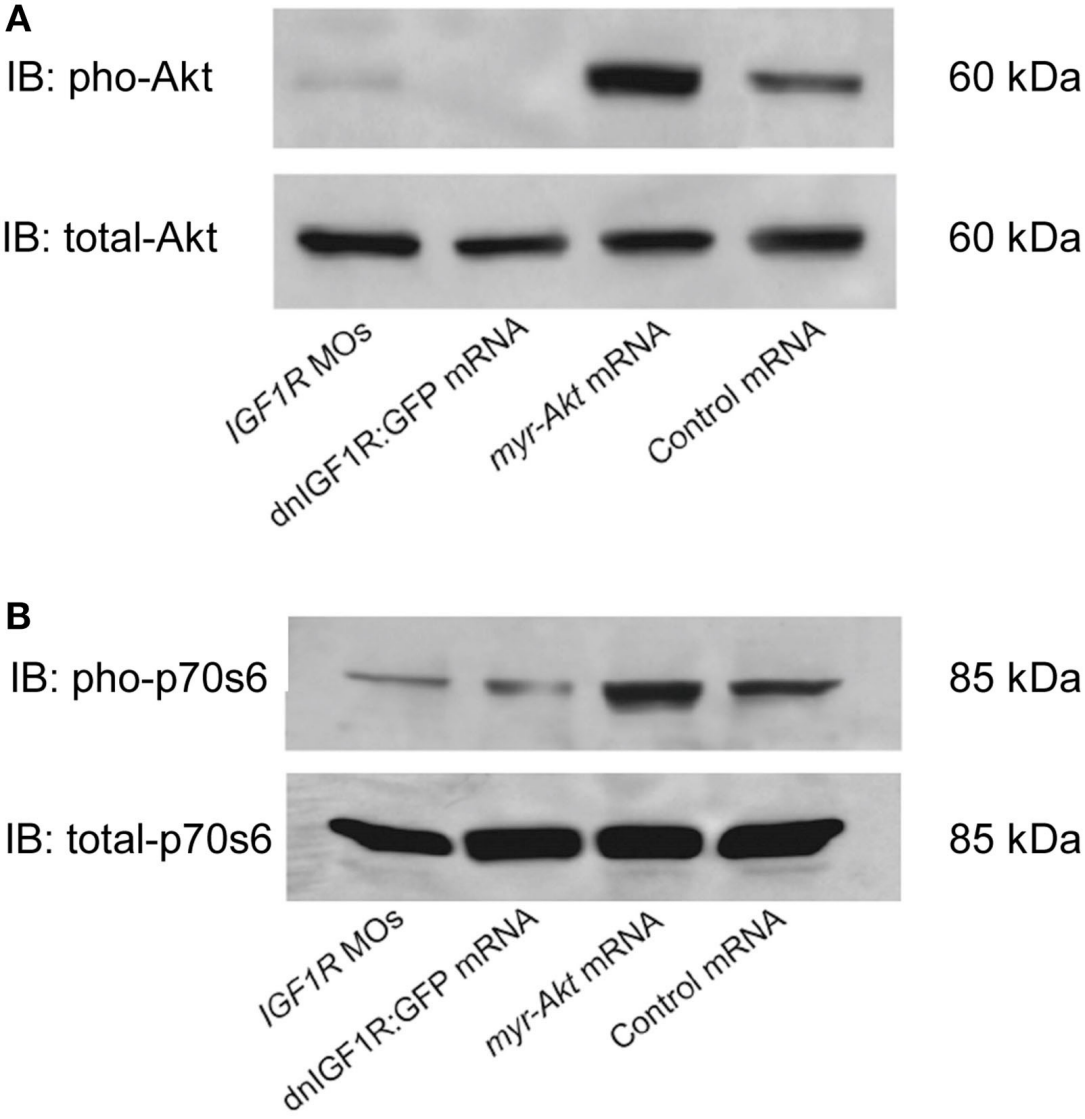
**Overexpression of myr-Akt increases levels of phosphorylated Akt and p70s6 kinase**. **(A)** Representative Western immunoblots using antibodies for phosphorylated and total Akt on embryos injected with IGF1R MOs, dnIGF1R:GFP mRNA, myr-Akt mRNA, or control mRNA (encoding GFP). **(B)** Western blot analysis of the embryos same as **(A)** using p70s6 kinase antibody. Note the increase in phosphorylated Akt and p70s6 kinase in myr-Akt mRNA-injected embryos compared to controls, and to embryos with reduced IGF1R-mediated signaling (IGF1R MOs and dnIGF1R: GFP). Total levels of Akt and p70s6 kinase protein are not significantly different among the treatment groups. Similar results were obtained in two other microinjection experiments.

An additional myristoylation sequence to Akt has been previously shown to be able to localize Akt to the plasma membrane where it can be more readily phosphorylated and rendered constitutively active (27–29). In order to test the effects of Akt overexpressing on zebrafish embryogenesis, considering the high degree of conservation, mRNA encoding mouse myr-Akt1 was transcribed *in vitro* and injected into embryos at the 1–2 cell stage. Injection of myr-Akt at concentrations above 200 pg mRNA per embryo caused severe and inconsistent morphological deformities (data not shown). As shown in Figure 2, overexpression of myr-Akt induced a significant increase of the levels of Akt and phosphorylation of its downstream molecule p70S6, comparing to controls and to embryos with reduced IGF1R-mediated signaling (IGF1R MOs and dnIGF1R: GFP). Therefore, these results provide *in vivo* biochemical verification that injection of myr-Akt caused increases in Akt and p70S6 kinase phosphorylation.

### PI3 Kinase Inhibition Modulated Brain Size Decrease Was Recovered by myr-Akt Overexpression

**Figure 3:**
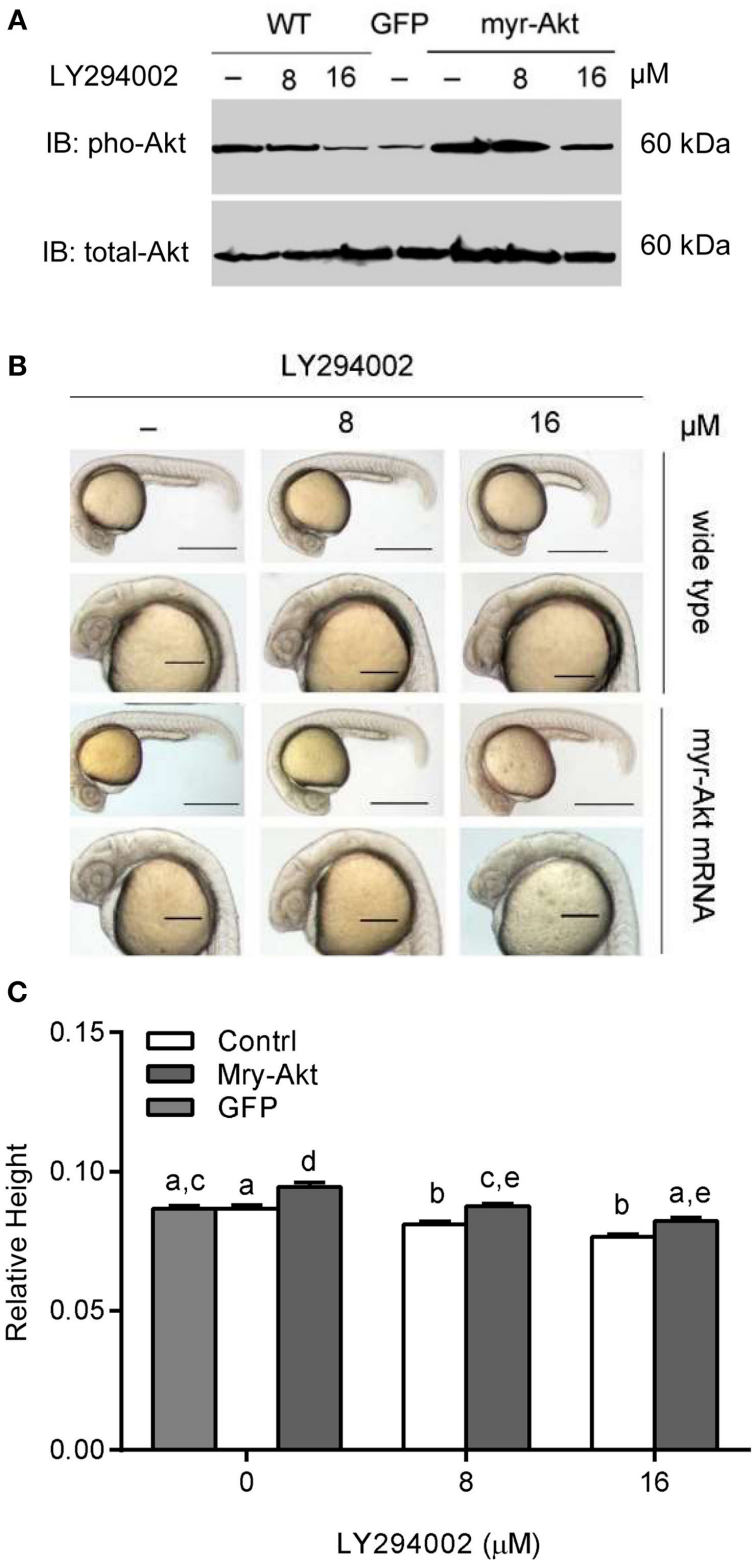
**Phosphoinositide 3-kinase inhibition associated brain size decrease was recovered by myr-Akt overexpression**. **(A)** Western immunoblots using antibody for phosphorylated Akt on embryos of wide-type and injected with myr-Akt mRNA treated with 8 µM, 16 µM, or without LY294002 or control mRNA (encoding GFP), with total Akt as a loading control. **(B)** Morphology of 24 hpf embryos of wild type and myr-Akt mRNA-injected group, treated with 8 µM, 16 µM, or without LY294002. GFP mRNA was injected as control mRNA. The first and third panel from the top, scale bar = 500 μm; the second and fourth panel displayed a close-up of the head region, scale bar = 200 μm. **(C)** Relative brain height of embryos with GFP or myr-Akt injected, treated with 8 µM, 16 µM, or without LY294002. Results are from three independent microinjection experiments, each with 25 embryos per group. *P* < 0.05 compared with the wide-type control group.

Akt is activated *via* receptor tyrosine kinases in a PI3K-dependent manner and phosphorylated on Thr308 and Ser473 by upstream kinases. We used western immunoblot to analyze the phosphorylation levels of Akt in embryos. The results illustrated that treating with a series concentration of LY294002 markedly reduced phosphorylation of Akt in embryos with 200 pg myr-Akt mRNA injection (Figure 3A) *via* blocking the PI3K function. As shown in Figures 3B,C, Akt overexpressing could partially rescue phenotypes of PI3K inhibition. Morphological inspection shows their brain size were obviously larger than those with LY294002 treatment. Furthermore, they appeared to be relatively normal comparing to wild type and GFP mRNA-injected embryos.

### Overexpression of myr-Akt Increases Brain Size in Zebrafish Embryos

**Figure 4:**
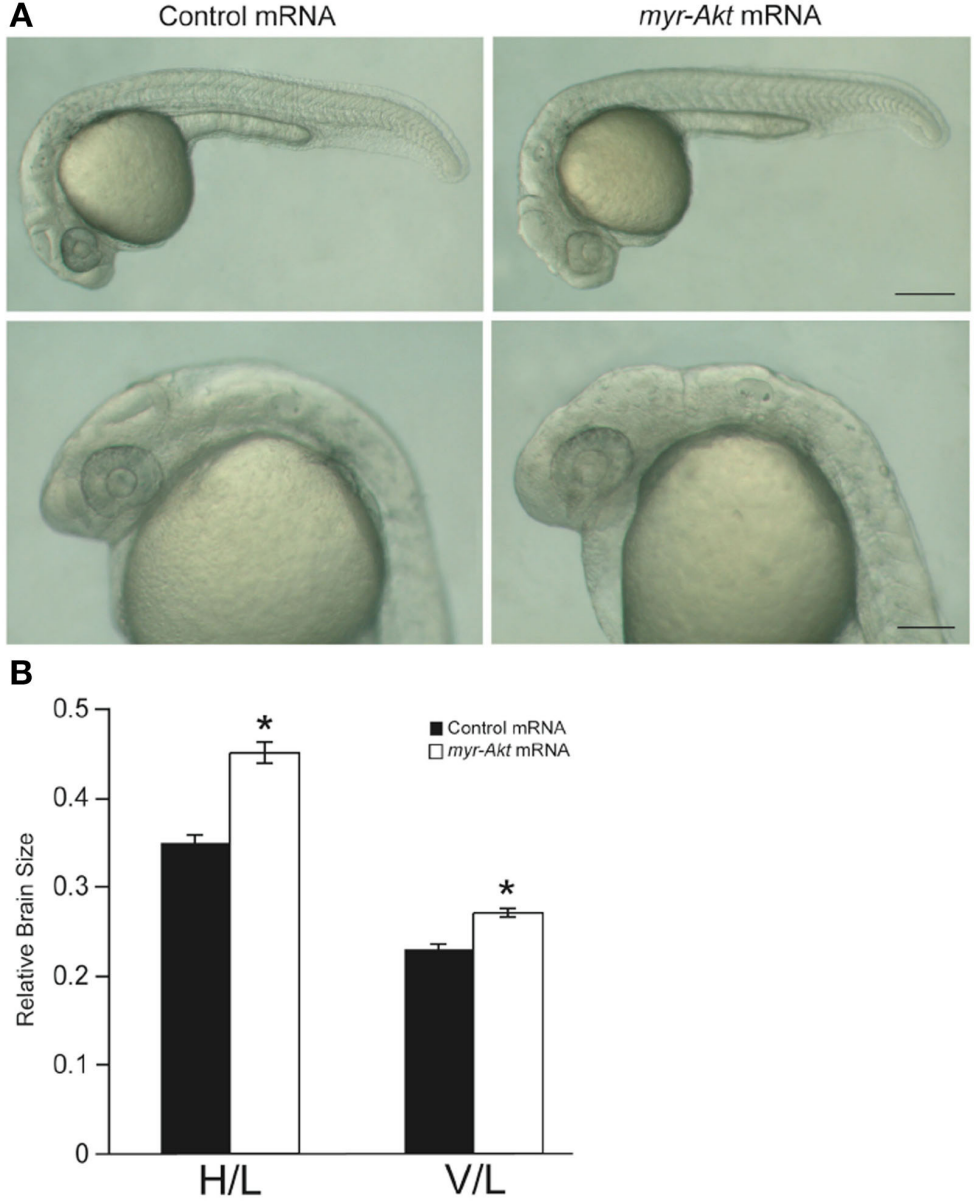
**Overexpression of myr-Akt increases brain size in zebrafish embryos**. **(A)** Morphology of zebrafish embryos injected with either control mRNA (left panels) or myr-Akt mRNA (right panels). Scale bar = 250 μm for top panels, and 100 µm for bottom panels. **(B)** Relative brain size measurements based on horizontal brain height (H) or vertical brain length (V), divided by inner ear to tail embryo length (L). Injection of myr-Akt significantly increased brain size compared to controls. *N* = 20 total embryos measured from two independent microinjection experiments, 10 embryos per group (*P < 0.0001).

At a concentration of 200 pg per embryo, injection of myr-Akt mRNA caused an increase in brain size in zebrafish embryos (Figure 4A), evident in ~77% of injected embryos at 24 hpf (*N* = 151 total embryos from three independent microinjection experiments). We observed a significant increase in relative brain height and length in embryos injected with myr-Akt mRNA compared to control mRNA-injected embryos (Figure 4B).

### Neuronal Cell Abundance, But Not Fate, Is Affected by Akt Overexpression through Apoptosis in the Brain

**Figure 5:**
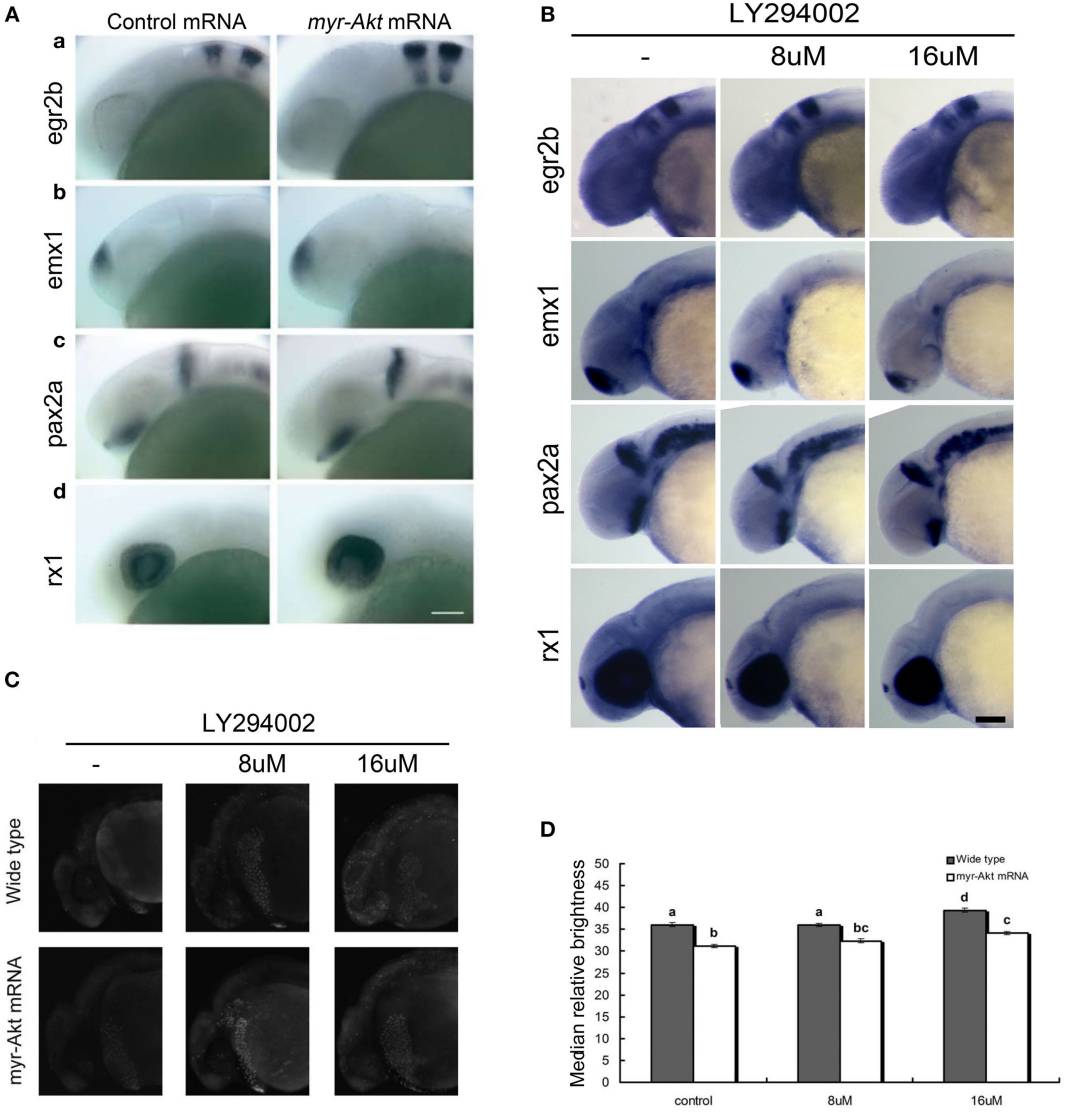
**Neuronal cell abundance, but not fate, is affected by Akt overexpression through influencing apoptosis**. **(A)** Representative whole mount *in situ* hybridization images of control mRNA (left panels) or myr-Akt mRNA-injected embryos (right panels) using probes for (a) egr2b (third and fifth rhombomeres of the hindbrain), (b) emx1 (forebrain), (c) pax2a (optic stalk, mid-hindbrain boundary), and (d) rx1 (retina). *N* = 9–13 embryos per group, all with similar expression patterns. Scale bar = 100 µm. **(B)** Whole mount *in situ* hybridization of embryos treated with or without LY294002 using probes as **(A)**. **(C)** Acridine orange staining of 24 hpf wild-type and myr-Akt mRNA-injected embryos treated without or with 8 µM, 16 µM LY294002. Scale bar = 100 μm. **(D)** Graphical representation of percentages of Median relative brightness of brain region after AO staining in each treatment group. *N* = 15 images quantified per treatment group. Groups with different letters differ significantly from each other (*P* < 0.05).

Since the embryos overexpressing Akt displayed abnormity mainly in the brain region during early developmental stage (24 hpf), the whole mount *in situ* hybridization was performed to analyze the expression pattern of several marker genes, egr2b (third and fifth rhombomeres of the hindbrain), emx1 (forebrain), pax2a (optic stalk, mid-hindbrain boundary), and rx1 (retina), involved in neural patterning of the brain (Figure 5A, a–d). In all cases, there is an increase in the expression domain of these genes, consistent with a larger brain size. In contrast, the expression domain of these genes decreased in embryos treated with LY294002 (Figure 5B). However, the localization of those neural markers in the embryos overexpressing Akt or treated with PI3K inhibitor was the same as that of wild-type embryos, suggesting that cell fate was unaffected. Thus, it indicates that overexpression of a constitutively active Akt in zebrafish embryos increases brain size but does not significantly alter brain patterning.

Next, we performed acridine orange staining on PI3K inhibitor-treated embryos of 24 hpf control and myr-Akt mRNA-injected embryos. As shown in Figures 5C,D embryos with myr-Akt mRNA injection had obviously lower numbers of AO-stained cells throughout the brain region. It revealed that PI3K inhibition-associated brain-size reduction might be realized *via* increased cell apoptosis.

### Constitutively Activated Akt Protects Neuronal Cell from Heatshock and UV Light-Induced Apoptosis

**Figure 6:**
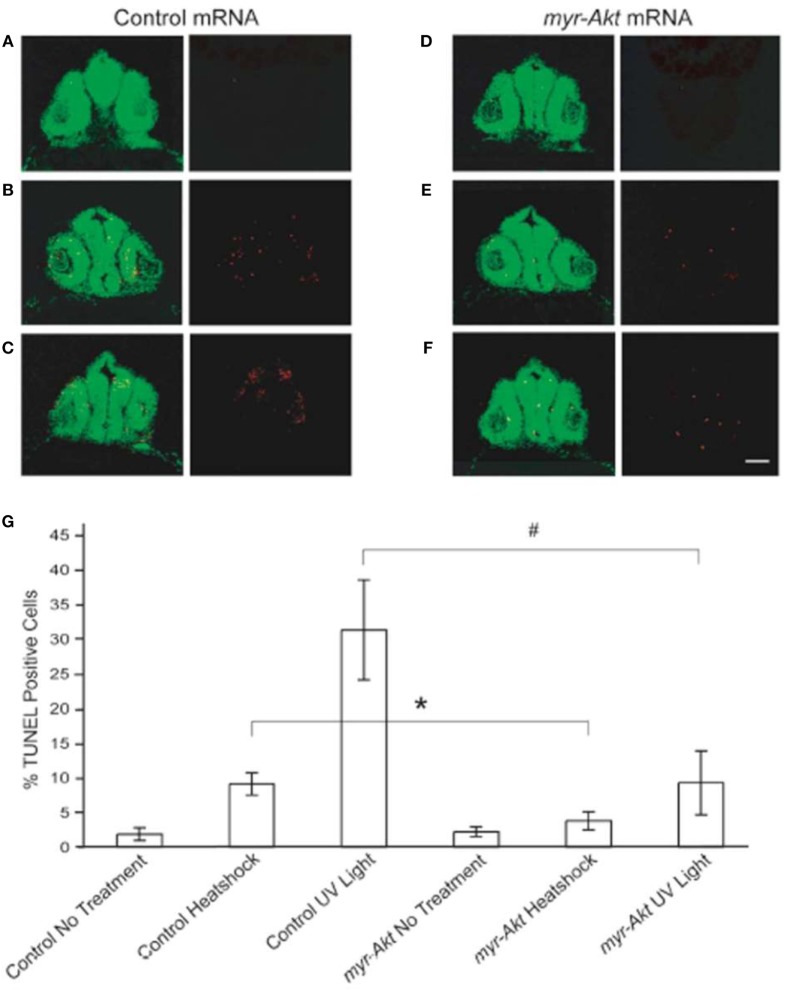
**Constitutively activated Akt protect neuronal cell from heatshock and UV light-induced apoptosis**. Representative images of cryosectioned control mRNA **(A–C)** or *myr-Akt* mRNA-injected embryos, **(D–F)** stained using TUNEL (red) and the nuclear dye, Sytox (green). Images on right side of panels are TUNEL only, while left side are overlaid images of TUNEL and Sytox staining. **(A,D)** Embryos without treatment; **(B,E)** embryos administered heatshock; **(C,F)** embryos administered UV light. Scale bar = 50 μm. **(G)** Graphical representation of percentages of TUNEL-positive cells in each treatment group. *N* = 6 images quantified per treatment group (*P < 0.05; ^#^*P* < 0.001).

Activation of Akt has been shown to be important for protecting neurons from apoptosis (18, 30). To test the ability of constitutively active Akt at protecting neuronal cell from apoptosis *in vivo* during zebrafish embryogenesis, we subjected control and myr-Akt mRNA-injected embryos to heatshock and UV light treatment. After administration of heatshock or UV light exposure at 20 hpf, embryos were allowed to continue to develop until 28 hpf and then fixed and stained for apoptotic cells using TUNEL. Compared to untreated control and myr-Akt mRNA-injected embryos, heatshock and UV light treatment showed significantly higher numbers of TUNEL-positive cells in the brains of control mRNA-injected embryos (Figures 6A–D,G). However, injection of myr-Akt mRNA significantly attenuated the heatshock and UV light-induced apoptosis (*P < 0.05; ^#^*P* < 0.001, respectively; Figures 6E–G). These results indicate that Akt overexpression in zebrafish embryos could inhibit neuronal apoptosis *in vivo*, and suggest that apoptotic inhibition may be part of the underlying causes of the increased brain size in untreated myr-Akt mRNA-injected embryos.

## Discussion

Phosphoinositide 3-kinase/Akt signal pathway has been shown to be essential for normal animal growth and development. Moreover, this pathway is found to be sufficient for trophic factor-induced neuronal survival and plays a significant role in central nervous system development. Trophic factors, such as nerve growth factor, IGF1, or brain-derived neurophic factor, lead to PI3K activation by binding to their cognate tyrosine kinase receptors (31–34). In our study, we presented data that PI3K inhibition led to brain size decrease *via* regulating apoptosis in the brain region, and overexpressing Akt successfully rescued the reduction of brain size in zebrafish early embryogenesis. Combined with the consistent phenotypes of PI3K inhibition in *Igf1^−/−^* mice (4) and IGF1R loss-of-function in zebrafish (35, 36), these results illustrate that PI3K/Akt mediate the effects of trophic factors on brain development and is also essential for embryonic brain development in zebrafish.

We also found a specific increase in brain size in zebrafish embryos overexpressing Akt1 mRNA at 24 hpf without displaying any ectopic expression. These results are also supported by the phenotypes of *Akt3* knockout mice, which exhibit an approximate 20% reduction in brain size (16, 37). Additionally, the following studies with brain-specific deletion of PTEN, which is an inhibitor for Akt phosphorylation, demonstrated brain enlargement in mice (38, 39). From the available data, we learn that Akt has a significant status in brain size regulation.

As we know, activation of Akt *via* the IGF1R is a particularly important survival-promoting signal during vertebrate development. The levels of IGF1R expression are directly proportional to the degree of apoptosis, with lower expression in cells that appear to be more susceptible to cell death (40, 41). Upon phosphorylation *via* Akt, the pro-apoptotic protein BAD is sequestered by the cytosolic protein 14-3-3 (42) and is unable to interfere with the anti-apoptotic protein BCL2 (43). Akt can also phosphorylate several pro-apoptotic forkhead transcription family members (44, 45) and thus prevent their activity by sequestration in the cytosol (46). This activity reduces the expression of several forkhead target genes, including the Fas ligand, causing decreased Fas-mediated apoptosis (47). From the results of acridine orange staining, we could tell that overexpressing Akt could protect the brain from apoptosis in zebrafish embryo. These results are consistent with earlier research that demonstrated knockdown of zebrafish Akt1 results in neuronal apoptosis (21). To test the effect of Akt overactivation on cell survival during zebrafish embryogenesis, we then induced apoptosis using heatshock and UV light treatment. We found that embryos expressing constitutively active Akt exhibited significant attenuation of both heatshock and UV light-induced apoptosis compared to control embryos. This suggests that overactivation of Akt can promote neuronal cell survival *in vivo*, which may be part of the mechanism underlying the increase in brain size during zebrafish embryogenesis.

Recent *in vitro* research also showed the protective roles of Akt in neuronal cells (19, 20, 22, 48). Other studies revealed that Akt2, but not Akt1, is essential for cell survival upon UV irradiation, and that Akt2 prevents UV-induced cell death by inhibiting activation of JNK and p38 in mouse embryonic fibroblasts and aortic endothelial cells (49). Additionally, Akt2 can resist apopotosis induced by oxidative stress through multiple signaling pathways including MDM2-p53-Bak, GSK3β-MCL-1, and FOXO3A-Bim (49, 50). Referring to research in mouse Akt3 knockout model and our results in zebrafish embryos, we found the conserved function among Akt isoforms in protecting neuronal cells. Future studies of Akt isoform-special function are important for elucidating Akt distinct roles during development *in vivo*.

These results provide a foundation for investigating the *in vivo* functions of Akt signaling during vertebrate brain development using the zebrafish as a model system. The proper function of Akt is required for vertebrate development, but the underlying cellular mechanisms by which Akt promotes cell growth and inhibits apoptosis *in vivo* are incompletely understood. Given its versatility as a model system, the zebrafish is uniquely positioned to provide novel insight into the embryonic functions of Akt. It will be important for future studies to identify and characterize the expression patterns of zebrafish Akt genes, as well as to perform functional analysis of their roles in embryogenesis.

## Author Contributions

SC, YLiu, and XR performed the research experiments. YLi and JZ participated in the data interpretation and helped to draft the manuscript. LL contributed to the design, supervision, and to the writing of the paper. All the authors read and approved the final manuscript.

## Conflict of Interest Statement

The authors declare that the research was conducted in the absence of any commercial or financial relationships that could be construed as a potential conflict of interest.
